# Combining Neural and Behavioral Measures Enhances Adaptive Training

**DOI:** 10.3389/fnhum.2022.787576

**Published:** 2022-02-14

**Authors:** Md Lutfor Rahman, Benjamin T. Files, Ashley H. Oiknine, Kimberly A. Pollard, Peter Khooshabeh, Chengyu Song, Antony D. Passaro

**Affiliations:** ^1^Department of Computer Science and Information Systems, California State University San Marcos, San Marcos, CA, United States; ^2^Human Research and Engineering Directorate, DEVCOM Army Research Laboratory, Los Angeles, CA, United States; ^3^Department of Computer Science and Engineering, University of California, Riverside, Riverside, CA, United States; ^4^DCS Corporation, Los Angeles, CA, United States

**Keywords:** electroencephalography, EEG, adaptive training, brain-computer interface, theta-alpha ratio, theta-alpha ratio percentage (TARP), behavioral adaptive training, combined adaptive training

## Abstract

Adaptive training adjusts a training task with the goal of improving learning outcomes. Adaptive training has been shown to improve human performance in attention, working memory capacity, and motor control tasks. Additionally, correlations have been observed between neural EEG spectral features (4–13 Hz) and the performance of some cognitive tasks. This relationship suggests some EEG features may be useful in adaptive training regimens. Here, we anticipated that adding a neural measure into a behavioral-based adaptive training system would improve human performance on a subsequent transfer task. We designed, developed, and conducted a between-subjects study of 44 participants comparing three training regimens: Single Item Fixed Difficulty (SIFD), Behaviorally Adaptive Training (BAT), and Combined Adaptive Training (CAT) using both behavioral and EEG measures. Results showed a statistically significant transfer task performance advantage of the CAT-based system relative to SIFD and BAT systems of 6 and 9 percentage points, respectively. Our research shows a promising pathway for designing closed-loop BCI systems based on both users' behavioral performance and neural signals for augmenting human performance.

## 1. Introduction

Training is a systematic approach to acquiring skills that improve performance in a task of interest. Training can be either non-adaptive or adaptive. A key assumption of training is that for any given skill level, there exists a difficulty of training that will provide the largest skill gains (Mané et al., 1989). A common kind of non-adaptive training uses a fixed difficulty level. Fixed difficulty training will be optimal only for individuals within a narrow range of skill. In adaptive training, the difficulty of the training is based on the trainee's performance to maintain an optimal difficulty range specific to the trainee. The main goal of the adaptive system is to set the level of task difficulty to the level that maintains the optimal level of learning for the trainee.

The impact of adaptive training over fixed training difficulty has been studied in the past, particularly in attention (Redick et al., 2013; Cuenen et al., 2016), working memory capacity (Klingberg et al., 2005; Jaeggi et al., 2008; Holmes et al., 2009; Karbach et al., 2015; Flegal et al., 2019), and motor control (Choi et al., 2008). Previous studies (Gopher et al., 1975; Park and Tennyson, 1980; Tennyson, 1981; Park and Lee, 2004; Romero et al., 2006; Graesser et al., 2012) showed that adaptive training is more effective compared to non-adaptive counterparts.

Although behavior-based adaptive training has had notable successes, behavioral data might be an incomplete indicator of optimal difficulty. Neurophysiological measures can provide additional information to adaptive systems, and electroencephalography (EEG) is one non-invasive way of measuring electrical potentials of the brain. For example, research has found a strong correlation between workload estimated from brain signals and task performance (Maurer et al., 2015; Dasari et al., 2017; Shoji et al., 2017). Incorporating neural signals into adaptive training systems may allow us to improve adaptive training beyond what can be achieved by incorporating behavioral measures alone.

The motivation to use EEG measurement compared to other modalities (e.g., GSR, eye movements, HR, and facial temperature distribution) was based on several reasons. First, EEG hardware is comparably cheap, has high temporal resolution and can detect brain responses within milliseconds of the stimulus presentation (Schneegass et al., 2019). Though it is difficult to find the best physiological indicators of workload, many studies showed EEG captures a more promising workload measurement than other indicators (Taylor et al., 2010; Hogervorst et al., 2014). Some users might prefer not to have a camera pointed at their face (eye movements, thermal imaging), and peripheral recording (GSR, HR) might not be specific enough to cognitive workload. Additionally, EEG is being incorporated into new mixed reality headsets, and companies like Facebook are working to integrate BCI-based technology. Our technology and research could inform industry and influence the way these kinds of headsets are used.

Previous studies (Gevins et al., 1997, 1998; Smith and Gevins, 2005; Lean and Shan, 2012) found that alpha power (EEG spectral power in the 8–12 Hz band) is related to overall attention such that as alpha power increases, focus and attention decrease. Theta power (EEG spectral power in the 3–7 Hz band) has been observed to fluctuate in the opposite direction in some cases so that a positive relationship between theta amplitude and focus exists. These two spectral bands fluctuate to varying degrees based on specific situations. An obvious approach would be to combine these two neural features as a ratio since they demonstrate negative (denominator) and positive (numerator) relationships with the behavior of interest (focus). Theta/alpha ratio (TAR) is one neural measure that showed promising results in closed-loop feedback systems for learning and several other cognitive tasks (Clarke et al., 2001; Egner et al., 2002; Egner and Gruzelier, 2004; Raymond et al., 2005; Koehler et al., 2009). However, TAR varies substantially from person to person based on the conductivity of their scalp, their anatomy, and their neural patterns. Here, rather than using absolute changes in TAR as the basis for neuro-adaptive training, we used relative TAR changes over time expressed as a percentage. We refer to this quantity as the Theta/Alpha Ratio Percentage, or TARP for details, see Section 2.6, Equation (1).

The relationship between brain activity and behavior as it is understood in the laboratory environment does not necessarily translate to real-world. We have created a technology that adapts training difficulty based on brain signals recorded from EEG. This technology may improve learning through a closed-loop brain-computer interface (BCI) that adaptively increases training difficulty based on pseudo real-time neural readings. In closed-loop BCI, the monitoring of brain activity can be used to set up the appropriate difficulty level in the training paradigm which eventually helps users to improve in subsequent transfer tasks. While previous studies (Rotenberg et al., 2013; Sitaram et al., 2017) describe the basic principles behind an online adaptive training system using different behavioral and neural metrics, here we designed and tested a closed-loop adaptive system whereby brain responses are monitored in real-time resulting in the modulation of training difficulty.

Behavioral features give insight into the relationship between cognitive workload and performance from one angle while neural measures may provide insight from another angle. Combining both features may yield a more accurate predictor of cognitive workload and its effects on performance. In this study, we explore the feasibility of adding neural features into behaviorally adaptive training to boost transfer task (A transfer task is a different task than the training task) performance. Specifically, we aim to answer the following question.

**Research Question** Will adding neural measures into a behaviorally adaptive training system improve human performance on a transfer task?

To answer the above question, we designed, developed, and conducted a user study that compared three training regimens: Single Item Fixed Difficulty (SIFD), Behaviorally Adaptive Training (BAT), and Combined Adaptive Training (CAT). The training methodologies differ only in how and whether the training difficulty changes. For the SIFD condition, task difficulty was fixed at the easiest level regardless of performance. For the BAT condition, task difficulty was varied based on the user's behavioral performance. For the CAT condition, task difficulty was varied based on both behavioral performance and neural measures. In all cases, the primary outcome of interest was performance on the transfer task.

Our results suggest that adding neural measures together with behavioral performance criteria leads to better learning. Results showed a statistically significant transfer task performance advantage of the CAT-based system relative to SIFD and BAT systems of 6% and 9%, respectively. These findings illustrate the promise of combining neural and behavioral features in practical applications of adaptive training.

## 2. Method

The goal of this study was to measure the effectiveness of adding neural measures within a closed-loop BCI into adaptive training. To achieve this goal, we designed three variants of a common training task, along with a shared transfer task. We also describe the participants, recruitment, apparatus, design, and procedure for conducting the experiment in this section.

### 2.1. Go/No-Go Training Task

**Figure 1:**
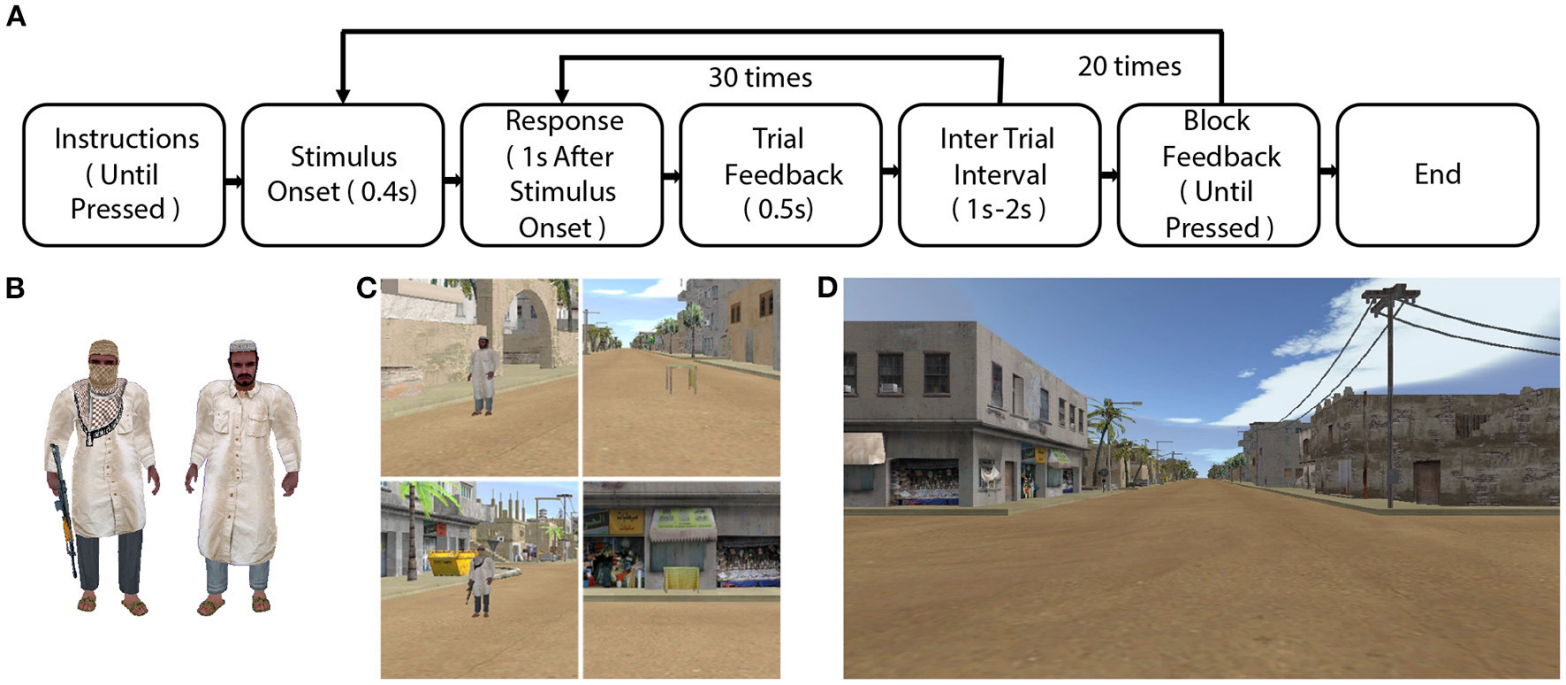
Method. **(A)** A flow chart of the training experiment. **(B)** Stimuli used in the go/no-go training task. **(C)** Trained (left column) and untrained (right column) threat (top row) and non-threat (bottom row) stimuli in the transfer task. **(D)** A screenshot from the transfer task.

The training task involved presentation of images (Figure 1B) that were designated as potential threats (a character holding a gun) vs. non-threats (a character without a gun). The participant's task was to respond to threats by pressing a button and to respond to non-threats by withholding a button press (go/no-go paradigm). This training was meant to improve inhibitory control in speeded perceptual classification of threats and non-threats. We designed our training task experiment based on a previous study (Files et al., 2019). The number of character(s) shown on the screen during each trial was dependent upon the training condition. For SIFD, there was only one character in each trial. For BAT and CAT, there were up to 5 characters depending on the adaptive logic for each trial. For any difficulty level of go trial, all character(s) were threats. For the no-go trials, one character was a non-threat. At difficulties two and greater, all other characters were threat characters. The characters were presented in random non-overlapping locations on the screen. The characters were computer-rendered images isolated from any background. The threat character was a male character holding a rifle and his face partially covered. The non-threat character was in similar attire but without a rifle and his face uncovered.

There were 20 blocks in the training task. Each block consisted of 30 trials. Thus, there was a total of 600 trials per participant in the training task. The ratio of go and no-go trials was 4:1. In each block, there were 6 no-go trials and 24 go trials. We pseudo-randomized the order of go/no-go trials with the constraint that there were no more than 7 go trials in between two no-go trials. Each stimulus was presented for 0.4 s on the screen. The participant had to respond within 1s after stimulus onset to register a response within the system. The trial feedback was given for 0.5 s after the response deadline. The duration of each trial was 1.5 s with a variable (uniform distribution) inter-trial interval of 1.0–2.0 s. The process flow diagram of the experiment is shown in Figure 1A.

In our experiment, a participant had to identify threats as quickly as possible by pressing a button using their dominant hand for threats (Go) and by withholding a button press (i.e., doing nothing) for a non-threat (No-Go). To measure participant performance, each trial was scored. There were up to 60 points assigned for go trials and 180 points assigned for no-go trials, with the participant earning more points for a faster response in go trials. They got the full 60 points if they respond within 170 ms, after which the points gradually decreased based on a piecewise-linear function of response time. The minimum 30 points was assigned for responses between 0.5 and 1.0 s. The participant got 180 points for correctly withholding a response for non-threat trial. No points were awarded if they responded after the deadline or responded incorrectly. We provided feedback indicating whether or not the participant was correct at the end of each trial by displaying a green check mark for a correct response and a red X for an incorrect response. The number of points received was not shown to the participant. The response marks for non-threat trials were four times larger than those for threat trials. From this common base, we built three versions of the training that differed in whether and when the difficulty of training changed. One used a single fixed difficulty, one adapted based only on behavior, and the third adapted based on a combination of behavioral and neural measures. The details of these versions follow.

#### 2.1.1. Single-Item Fixed Difficulty (SIFD)

In the SIFD condition, task difficulty was fixed at the easiest level (no more than one character on the screen at any given time), regardless of the user's performance. SIFD is a non-adaptive training methodology. The SIFD condition data was acquired from a previous experiment (Files et al., 2021) with task parameters, equipment, and stimuli identical to the BAT/CAT conditions. The SFID condition represented a conventional approach to oddball training, and it served as a reasonable alternative to a no-contact control. Other controls could be used in future work; however, we believe that the SFID condition presents an adequate baseline to which we can compare performance.

#### 2.1.2. Behavioral Adaptive Training (BAT)

In the BAT condition, we changed the number of characters shown on the screen during a trial based on the user's score on the preceding block. The theoretical motivation for our BAT condition comes from well-supported theories in expertise development, education, and game design. In the study of expert performance, *deliberate practice* has been identified as an important contributor to attainment of expertise (Ericsson et al., 1993). Deliberate practice entails repetition with the focused intention to improve with an appropriately set goal. An appropriate goal is one that targets a difficulty level just beyond the current mastery so that practice challenges the user rather than calcifying behaviors that only suffice at the current level of difficulty. Relatedly, the instructional process of *scaffolding* (Wood et al., 1976) entails providing sub-goals and activities that are within the trainee's capabilities while challenging them to learn and grow those capabilities. Finally, within game design is the concept of a *flow zone* (Chen, 2007), in which a game is not too challenging and not too simple in order to maintain a state of focused engagement. The common thread among these theories is that appropriately selected difficulty is a critical element in effective practice. To maintain an appropriate level of difficulty, we developed a behaviorally adaptive training rule in which difficulty would increase once the trainee achieved criterion performance at the current difficulty (Table 1, Top). The criterion was derived based on average performance in the SIFD condition. To ensure that difficulty was not set too high, difficulty was reduced if performance approached what could be obtained by thoughtless responding (i.e., responding as quickly as possible regardless of the presence or absence of a target).

**Table 1:** Adaptive logic.

**BAT**			
Behavioral →	S <1500	1500 < =S < =1700	S>1700
	-	=	+
**CAT**			
Behavioral →	S <1500	1500 < =S < =1700	S>1700
Neural ↓			
TARP < -5	-	=	=
-5 < =TARP < =5	=	=	=
TARP>5	=	=	+

*Top: Behavioral-based adaptive logic for the BAT system. Bottom: Behavioral and neural based adaptive logic table for the CAT system. The difficulty level increased (+) by one level, decreased (-) by one level, or remained the same (=). Blue cells show when the behavioral component recommends a change that is not accepted by CAT*.

#### 2.1.3. Combined Adaptive Training (CAT)

In the CAT condition, task difficulty was varied based on both the behavioral scores and EEG values. The neural point thresholds of -5 and +5% used in CAT were based on pilot data from two users. The difficulty level was adjusted based on the adaptive logic presented in (Table 1, Bottom). This scheme incorporates both the neural and the behavioral measure. The adaptive logic was applied after the second block, where we got the first percentage of change of TAR value.

### 2.2. Target Identification Transfer Task

After training, participants engaged in a transfer task (Figures 1C,D) that has been used previously (Metcalfe et al., 2015; Files et al., 2019). This transfer task involves the same perceptual judgment of threat and non-threat as in training, but it takes place in a naturalistic context and with both trained and untrained stimuli. The context was a simulated 10-min ride in a vehicle in a semi-realistic virtual desert village environment. The untrained stimuli were tables with or without a cloth obscuring the view beneath the table. Unobscured tables were non-threats, and obscured tables were threats, because they might conceal a threat.

The transfer task stimuli were static 3D models added to the environment. There were 200 stimuli belonging to the four stimulus categories. The stimuli appeared in randomized order. The stimulus distance from the center was randomized. The vehicle speed was fixed. The participants saw each stimulus over a range of sizes and angles. We added an additional level of difficulty by displaying an intermittent fog (30 s to 2 min) five times throughout the transfer task. During the task, one of the four stimuli appeared on the screen randomly and stayed for 1 s. The inter-stimulus interval was uniformly distributed within 1–3 s. The participants had to press either one of two buttons within the 1-s response window. They responded to threats (Human, Table) stimuli with their dominant hand (based on the Edinburgh Handedness Inventory test) and to non-threats (Human, Table) stimuli with their other hand. We provided feedback with a green letter Y for the correct response, red letter N for incorrect response, and white letters OO for non-response. We evaluated users' performance by measuring the accuracy of detecting the objects (threat/non-threat) within the transfer task.

### 2.3. Participants

We recruited 68 healthy adult participants *via* Craigslist. Subjects were screened for normal or corrected-to-normal binocular vision (minimum of 20/40 acuity) using a standard Snellen chart and for color vision using a standard Ishihara 14-plate color test. Individuals were excluded if they reported a tendency for motion sickness or any brain-related diseases.

One participant failed the vision test. We could not use data from 12 participants (SIFD = 2, BAT = 6, CAT = 4) due to technical failures with the equipment and 11 participants (SIFD = 2, BAT = 6, CAT = 3) who failed to respond to transfer task stimuli more than 80 responses out of 200 responses. This left 44 total participants: 22 (M 13, F 9, mean age 29.40, SD 11.6) in the SIFD condition, 10 (M 4, F 6, mean age 33.4, SD 8.7) in the BAT condition, and 12 (M 5, F 7, mean age 29.83, SD 5.5) in the CAT condition. All participants gave voluntary, fully informed, and written consent to participate in our study. We maintained standard procedure for the study protocol approved by the Army Research Laboratory Institutional Review Board (IRB) under protocol number 17–166.

### 2.4. Apparatus

#### 2.4.1. Dell Ultra Sharp 24” Desktop Monitor

We used a 24” standard desktop monitor, keyboard, and mouse to execute the tasks for our study. It was built and manufactured by Dell.

#### 2.4.2. ActiveTwo Biosemi System

We recorded EEG using an ActiveTwo Biosemi system (BioSemi, Amsterdam, The Netherlands), which is a research-grade multi-channel, high-resolution biopotential measurement device. It has an electrode cap with 64 pre-amplified, active surface electrodes. The sensors were placed into the cap holes in accordance with a modified 10–10 electrode placement system (Jurcak, Tsuzuki, & Dan, 2007). A water-soluble, saline gel was inserted into the cap holes before placing the electrodes to ensure better connectivity with the scalp surface and electrodes. There are four additional sensors placed onto the skin for monitoring EOG related eye movements. The Common Mode Sense (CMS) electrode and Driven Right Leg (DRL) electrode served as a ground. The sampling rate for our experiment was 512 Hz. The EEG signals captured through active electrodes and were then converted to digital format using an AD-box (Analog to Digital box). The digitized data were then sent to a PC/Laptop. The Biosemi provides a proprietary software interface named ActiView for data processing.

#### 2.4.3. Instrument Control Toolbox

We used an Instrument Control Toolbox for TCP/IP communication between Matlab instance in stimlus PC and ActiView in Biosemi data recording PC. Here, ActiView software configured as TCP/IP server and one of the Matlab instance in stimulus PC configured as TCP/IP client. The Matlab program parsed incoming data packets, and stored the data from all channels.

### 2.5. Procedures

In this step, the devices were prepared for starting the training and testing session. Participants were fit with a 64-channel cap using a modified 10–10 electrode placement with external electrodes placed at the external canthi of the eyes, above and below the left eye, and on the mastoids. Once the cap was on the participant's head, the participant was asked to fill out the questionnaires on a laptop. The operator was gelling each electrode while the participant was completing the questionnaire. The participant was then taken into a soundproof room after the completion of sensors preparation and questionnaire. The operator then calibrated the EEG laptop in ActiView software interface. Electrode offsets were maintained at or below 30 uV. The participant was told to read all the on-screen instructions carefully during the whole duration of the experiment. The participant was instructed to maintain consistent eye distance to the monitor and to keep their legs uncrossed and both feet on the floor. The participant had the chance to ask any questions for clarification. The operator then closed the soundproof room door and turned on a video camera for observation and a microphone to speak with the participant if needed.

#### 2.5.1. System Setup

We used two computers for the experiment: the stimulus computer and Biosemi data recording computer. Two Matlab instances were running on the stimulus computer. One instance was for displaying the stimulus, and other instances were for communicating with Biosemi ActiView via TCP/IP connection. We used PsychToolBox (PTB) as our stimulus presentation software. During the training task, PTB sends the stimulus onset trigger signals to the Biosemi equipment simultaneously using Biosemi USB Trigger Interface Cable. The delay of Biosemi USB Trigger Interface Cable is less than 200 microseconds. A photodiode was installed into the bottom left corner on the stimulus onset computer for accurate timing of stimulus onset. The participant responded to the go task using an RT-box installed connected with stimulus onset computer.

#### 2.5.2. Task Execution Session

After the preparation session, the training task started and lasted for an average of 30 min followed by the participant filling out a questionnaire. The transfer task started with the completion of that questionnaire set. The duration of the transfer task was always fixed at 10 min. By the end of the transfer task, there was an exit questionnaire.

### 2.6. EEG Data Processing

We recorded EEG data using the 64-channel high-end Biosemi data acquisition system and used MATLAB (version-9.1.0.441655 R2016b) to analyze the EEG data. The sampling rate was 512 Hz. We received the continuous EEG data from the ActiView Biosemi software device over TCP/IP channel. We synchronized the stimulus and its EEG recording using a photo-diode in the USB2 receiver. We performed a column-wise Fast Fourier Transform across all 64 channels to calculate the theta (3–7 Hz) and alpha (8–12 Hz) power of the EEG in the 1s following the stimulus onset. We computed the ratio of theta to alpha for each electrode, and these ratios were averaged over all channels to obtain a single-trial whole-head TAR value. These single-trial TAR values were averaged over all trials in a block to obtain a block-level TAR. We then calculated the percentage change in block-level TAR from the previous block using Equation (1). We refer to this theta/alpha ratio percentage change as TARP.


(1) $$\begin{array}{r} TARP=\frac{TAR_{block\left(i\right)}-TAR_{block\left(i-1\right)}}{TAR_{block\left(i-1\right)}}*100 \end{array}$$

For offline processing, continuous data were high-pass filtered at 1.0 Hz, low-pass filtered at 40.0 Hz, average referenced, and then submitted to ICA with PCA reduction to 40 dimensions using standard PICARD with up to 500 iterations (Ablin et al., 2018). The resulting independent components were classified using ICLabel v.1.2.2, (Pion-Tonachini et al., 2019) and components classified as having a greater than 0.7 of belonging to a non-brain class (Muscle, Eye, Heart, Line Noise, Channel Noise, or Other) were removed from the data. Data thus cleaned were submitted to TAR and TARP calculation. Data from 4 participants (1 control, 3 CAT) were not usable for offline re-analysis due to errors during saving.

## 3. Results

**Figure 2:**
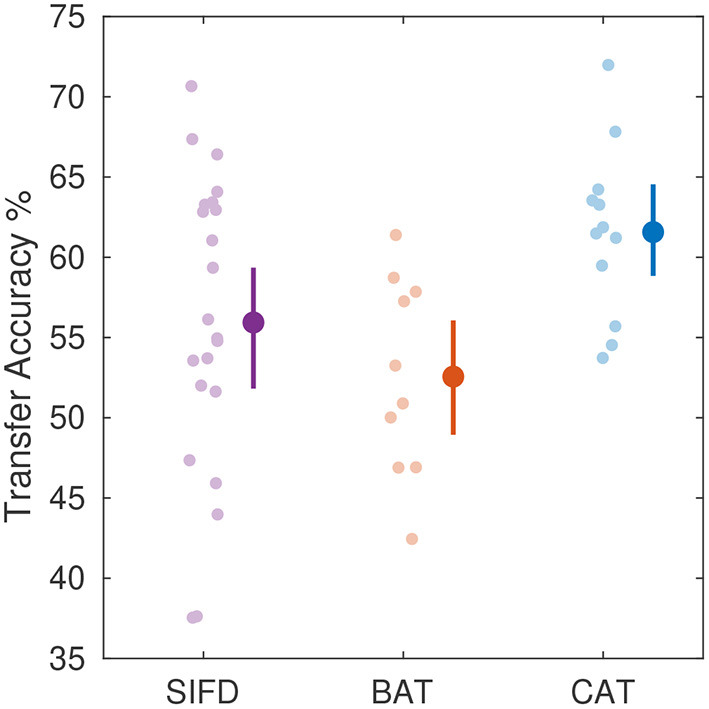
Average transfer task performance for the three training conditions. The combined adaptive training (CAT) resulted in better transfer task performance compared to behavior adaptive training (BAT) and fixed difficulty (SIFD) conditions. The large dots show the sample average, and the small dots show individual data points. Error bars show 95% confidence intervals from bias corrected, accelerated percentile bootstrap.

The main goal of this study was to measure the effectiveness of adding neuro-adaptivity into adaptive training. As a measure of training effectiveness, we examine transfer task performance. The different training conditions varied in difficulty, so comparisons of performance during training are less meaningful. Specifically, we evaluated the effectiveness of our training regimens by measuring the accuracy (percentage of the correct response) of the post-training transfer task. In this study, we had three separate training tasks (one for each condition) along with a shared transfer task. Average transfer task accuracy for each condition is shown in Figure 2.

The average transfer task accuracy scores in the SIFD, BAT, and CAT conditions were 55.93, 52.56, and 61.57%, respectively. The CAT system improved transfer task performance 10% relative to SIFD (6% point improvement) and 17% relative to BAT (9% point improvement). We conducted two sample *t*-tests with unequal variance adjustment and corrected *p*-values for multiple comparisons using the Benjamini and Hochberg (1995) procedure for controlling false discovery rate (FDR) to be 0.05. The results indicated a statistically significant advantage of the CAT condition over the SIFD condition, *t*(31.85) = -2.25, *M* = -5.63 percentage points, 95% CI [-10.7, -0.5], *p* = 0.047. There was also a statistically significant advantage of the CAT condition over the BAT condition, *t*(17.98) = -3.63, *M* = -9.0 percentage points, 95% CI [-14.2, -3.8], *p* = 0.006. Accuracy under BAT was lower than under SIFD, but this difference was not statistically significant, *t*(25.48) = -1.21, *M* = -3.4 percentage points, 95% CI [-9.1, 2.3], *p* = 0.236. These results show that participants in the CAT training condition performed better on the transfer task than those in the BAT and SIFD conditions.

**Figure 3:**
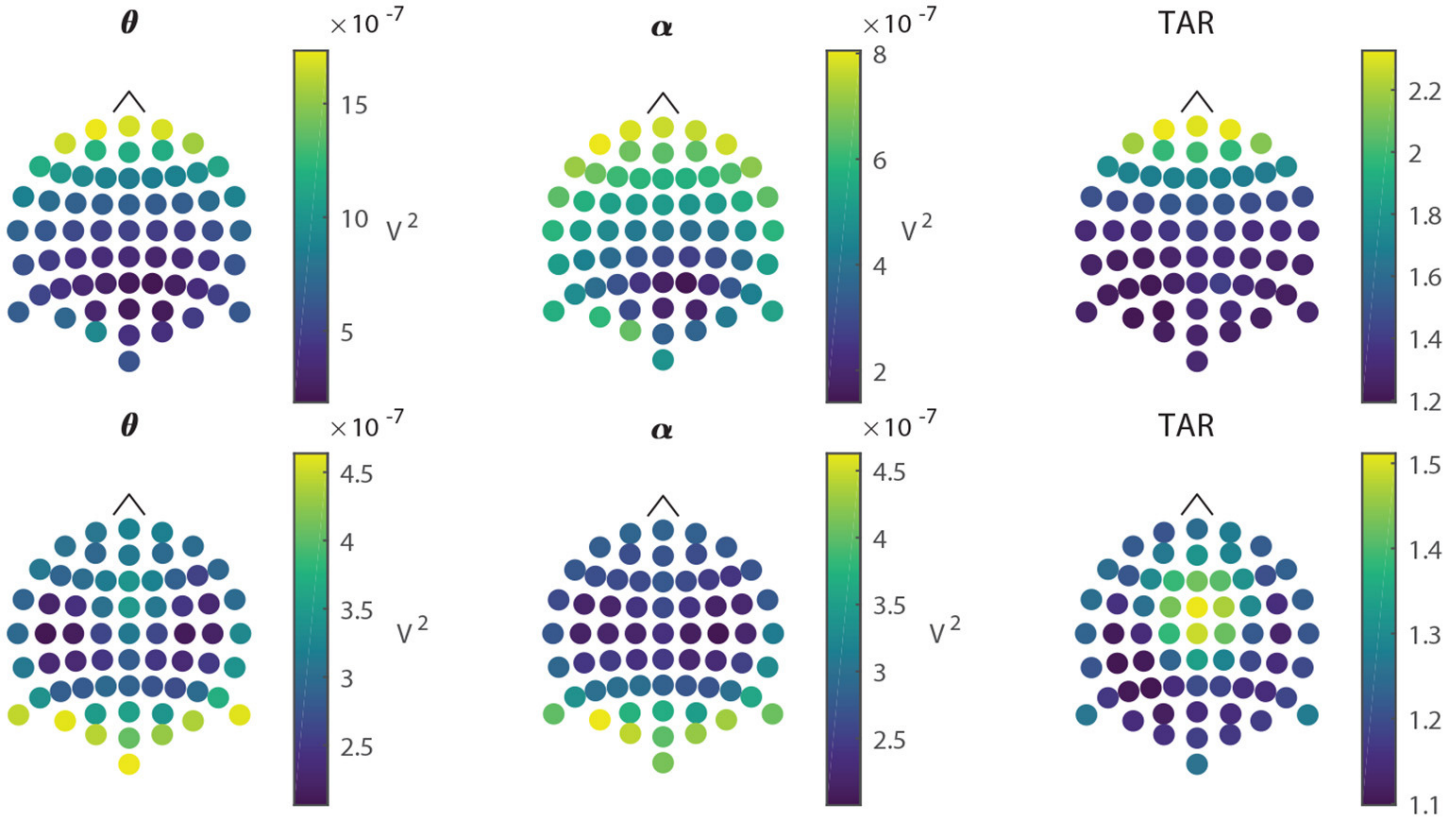
Scalp maps of online and offline data. The upper row shows scalp maps (one dot per electrode) for theta-band power, alpha-band power, and TAR (left-to-right, respectively) from the original data. The lower row shows the same maps computed from the offline data after artifact rejection. Note the differences in scale.

Group-average scalp maps of alpha power, theta power, and TAR appear in Figure 3, top row. The online calculation of TAR included raw EEG data from all sensors using no special filtering or data exclusion. This potentially included eye, muscle, and other artifacts. To better understand the impacts of those artifacts, we conducted an offline re-analysis of the EEG data. Group average scalp maps of alpha power, theta power, and TAR for the offline re-analysis appear in Figure 3, bottom row. To assess the effect of artifact rejection on TARP, we computed TARP for all blocks (regardless of experimental condition) both with and without artifact rejection. The correlation between TARP with and TARP without artifact cleaning was *r*(778) = 0.67. Considering only the participants in the CAT condition and only on those blocks in which the behavioral score was in a range that would permit a difficulty change (i.e., only those blocks in which the TARP had a unique effect on the difficulty change), offline and online TARP agreed in 83.8% of cases. Although we cannot know how transfer task performance might have been affected if difficulty changes followed the recommendations of the offline reprocessed data rather than the online, we view the high agreement as indicating the TARP was not dominated by artifacts.

In what follows, we examine some possible explanations for the transfer task performance advantage of CAT relative to BAT and SIFD. The CAT condition training was the only one to include a neuro-adaptive training rule. This led to differences in the training experienced by participants assigned to the CAT condition. Here, we examine whether differences in training difficulty, training performance, or difficulty stability could account for the CAT advantage. The general approach is to look at whether controlling for difficulty, performance, or stability eliminates the performance advantage of training under the CAT condition.

**Figure 4:**
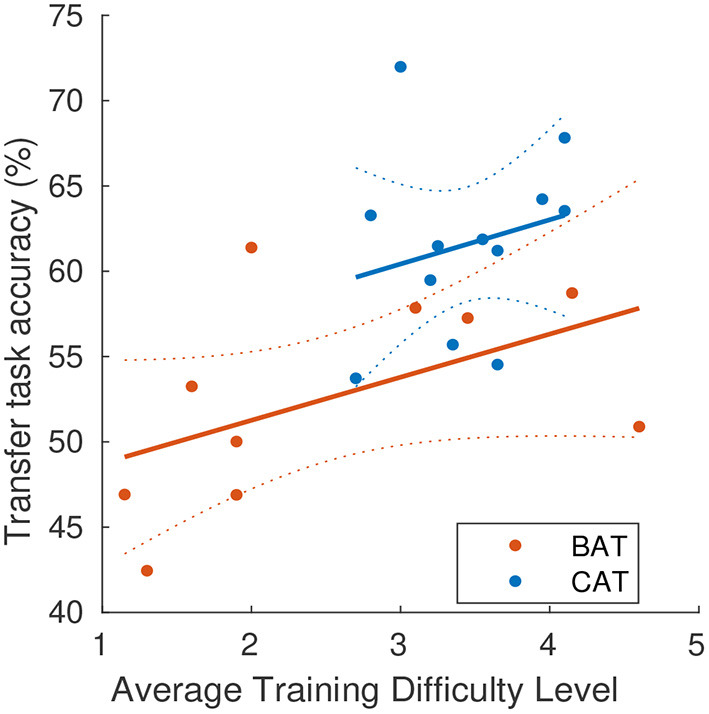
Differences in training difficulty do not account for the transfer performance advantage of CAT. Here, we excluded SIFD condition as it was fixed at easiest level. Dashed lines show 95% confidence intervals of the fit.

**Training difficulty level does not account for CAT's advantage on transfer task performance**. We observed that the CAT regimen led to a greater average difficulty level during training, as compared to the BAT. This suggested that perhaps training at higher difficulty accounts for the transfer task advantage of CAT. If difficulty level fully accounts for transfer task performance, then a model with training difficulty and training condition as predictors would be expected to find a significant coefficient of difficulty level and a condition coefficient to be close to zero. To examine whether the difference in average difficulty could account for the better transfer task performance with CAT training, we fit a linear model (Figure 4) with transfer task accuracy as the outcome variable and average training difficulty level and training condition as predictors. Condition was dummy-coded with BAT as the reference level. Continuous variables were mean-centered prior to model fitting. Training in the CAT condition was associated with a transfer task accuracy improvement of 6.64, 95% CI [0.55, 12.72] percentage points, *t*(18) = 2.29, *p* = 0.034 relative to training in the BAT condition. The effect of difficulty level in the BAT condition was 2.53, 95% CI [-0.64, 5.70] percentage points per level, *t*(18) = 1.67, *p* = 0.11. The interaction of condition with difficulty level, representing the difference in the effect of difficulty level in CAT relative to BAT was 0.07, 95% CI [-7.96, 8.09] percentage points per level, *t*(18) = 0.02, *p* = 0.99. There was a marginal association between difficulty of training and transfer task accuracy, but the transfer task performance advantage associated with CAT remained when controlling for this marginal effect.

**Figure 5:**
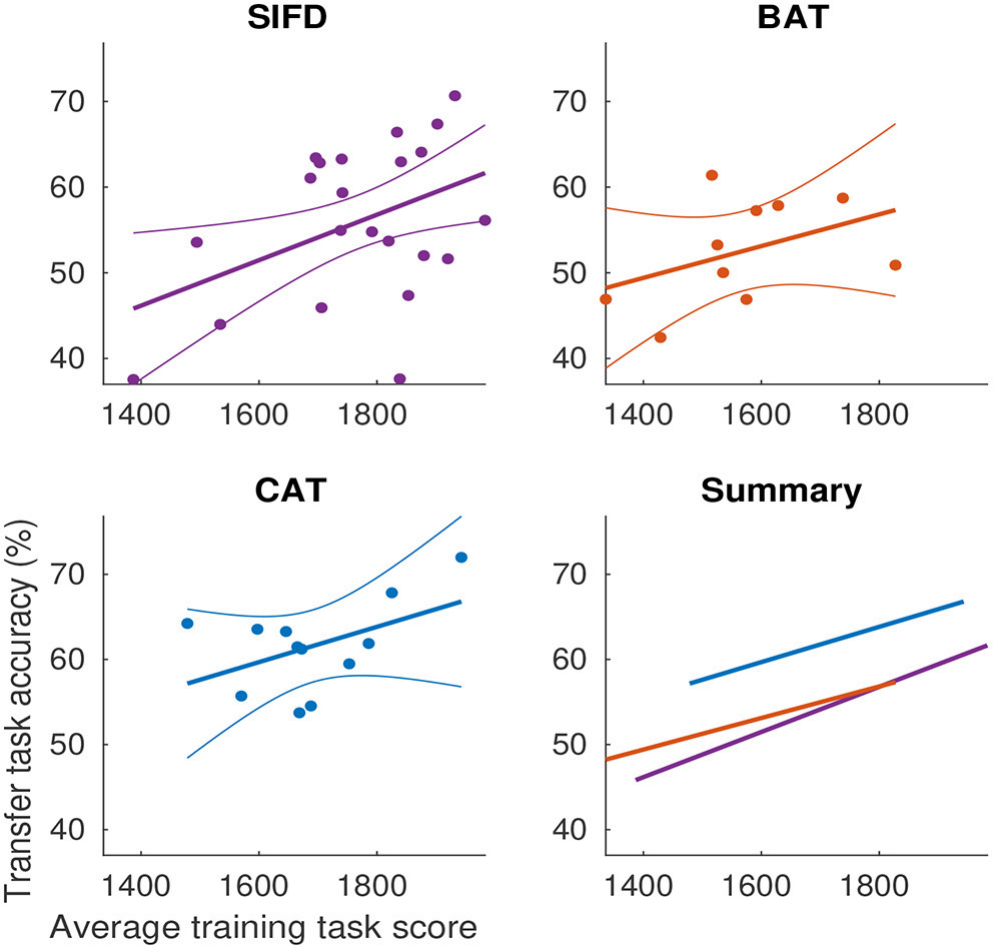
Differences in training task scores do not account for the transfer performance advantage of CAT. Curved lines show 95% confidence intervals of the fit.

**Training score does not account for CAT's advantage on transfer task performance**. We also observed differences in training task performance among the different training conditions. To examine whether the difference in average training task score could account for the transfer task accuracy advantage associated with CAT, we fit a linear model (Figure 5) with transfer task accuracy as an outcome variable and average score and condition as predictors. Condition was dummy-coded with SIFD as the reference category. At an average score, BAT increased accuracy by 0.82, 95% CI [-6.55, 8.20] percentage points, *t*(38) = 0.23, *p* = 0.82. CAT increased accuracy by 7.6, 95% CI [2.24, 13.09], *t*(38) = 2.81, *p* = 0.008. The effect of a one point increase in average score on accuracy was 0.03, 95% CI [0.005, 0.048] percentage points, *t*(38) = 2.48, *p* = 0.018. The interaction of BAT with score was -0.008, 95% CI [-0.05, 0.03], *t*(38) = -0.40, *p* = 0.69, and with CAT was -0.006, 95% CI [-0.05, 0.04], *t*(38) = -0.28, *p* = 0.78. This demonstrates that higher training task score was associated with better transfer task performance, but the transfer task performance advantage of the CAT condition remained when controlling for training task score.

**Training difficulty stability does not account for CAT's advantage on transfer task performance**. In the CAT condition, difficulty changes were less frequent compared to the BAT condition. This was a consequence of the construction of the BAT and CAT difficulty change logic. Table 2 summarizes the percentages of blocks that triggered the rules to increase, decrease, or keep difficulty the same. The table also highlights blocks in the CAT condition where the BAT rules would have triggered a difficulty change but the CAT rule did not (i.e., CAT was stabilizing, 50.4% of blocks).

**Table 2:** Percentage of block scores in adaptive logic categories.

**BAT**			
Behavioral →	S <1500	1500 < =S < =1700	S>1700
Total % of block	37.00	28.00	35.00
**CAT**			
Behavioral →	S <1500	1500 < =S < =1700	S>1700
Neural ↓			
TARP < -5	6.25	6.25	12.95
-5 < = TARP < = 5	6.70	15.18	25.89
TARP>5	4.90	7.59	14.29
Total % of block	17.85	29.02	53.13

*Top: Overall percentage of scores (S) in the three categories defined by the adaptive rule in the behavior adaptive condition. Bottom: Overall percentage of scores in the nine categories defined by the adaptive rule in the combined adaptive condition. Blue cells show when the behavioral component recommended a change that was not accepted by CAT*.

To check whether this stabilizing effect might account for the difference in transfer task accuracy, we ran a simple linear model with the number of novel blocks as the only predictor. The effect of the number of novel blocks on accuracy was marginal, *B* = -0.78, 95% CI [-1.60, 0.03], *t*(20) = -2.0, *p* = 0.059. A linear model with condition, number of novel blocks, and their interaction found a significant effect of the CAT condition, *B* = 9.23, 95% CI [2.01, 16.46], *t*(18) = 2.69, *p* = 0.015, but the effect size of number of novel blocks and the interaction were not statistically significantly different from zero (both *p* >0.2). However, the strong correlation between the number of novel blocks and condition (*r*(20) = 0.69) makes these coefficients difficult to interpret. These results show that the effect of difficulty stability did not have a major impact on transfer task performance, and controlling for stability did not eliminate the transfer task advantage of the CAT training condition.

## 4. Discussion

We developed a multi-modal adaptive training system by combining both behavioral and neural features and compared it with a non-adaptive and a behaviorally adaptive training system. We found that compared to the non-adaptive and the behavior-adaptive conditions, the combined neuro- and behaviorally adaptive training yielded significantly improved target classification performance on a near transfer task. In follow-up analyses, we considered some possible explanations for the advantage of CAT relative to BAT and SIFD conditions on the transfer task.

The different difficulty adjustment rules led to different average training difficulties, different training task scores, and different frequencies of difficulty change among the three conditions. Average training difficulty and training task score were both positively associated with transfer task performance, but these associations by themselves were not sufficient to explain the advantage of the CAT on transfer task performance.

One major effect of adding the neural component was to stabilize the difficulty level of training. In 50.4% of blocks, the CAT system over-rode the behavioral component's recommendation to change difficulty, so difficulty was more stable under CAT compared to BAT. However, we did not find convincing evidence that the frequency of difficulty changes during training was associated with better or worse performance on the transfer task. Based on this observation, adjusting the behavioral rule to recommend changes less frequently seems unlikely to reproduce the transfer task performance advantage of CAT.

We find no evidence supporting the possibility that the advantage of CAT is due to some artifact of the construction of the task or rule, leaving us with the explanation that the neural features captured in the CAT usefully supplemented the behavioral adaptation rule to maintain a task difficulty that promoted more successful learning. In what follows, we consider the theoretical implications of using percentage change in theta/alpha ratio as a neural feature, the practical implications of combining neuro- and behaviorally adaptive training, and future directions for related work.

**Value of neural features**. The difficulty change rules in the BAT condition were designed to keep the training at a difficulty level that was not too easy to be disengaging but not too difficult to be overwhelming. This choice was motivated by theoretical accounts that posit optimal difficulty selection is key to learning and engagement. However, training task performance is not necessarily a perfect indicator of the workload or mental effort the trainee was under. In other words, subjective difficulty does not necessarily align with objective difficulty. This suggests an explanation for the advantage of CAT over BAT, that it provided a more detailed indication of the subjective difficulty of training.

It is possible that a behaviorally adaptive rule could be designed that captured a better indicator of subjective difficulty. Our results suggest that average difficulty, average performance, and difficulty stability are not promising targets for a better behaviorally adaptive rule. If such a rule were developed, it would create an opportunity to test whether the neural features used here no longer improved training effectiveness.

**Implications and Applications of Our Work**. We believe our study advances the field in a few ways. Our method is, especially applicable in learning studies, because it is based on estimating percentage change in the theta/alpha ratio, rather than estimating an absolute objective or subjective workload level. Also, our EEG measure requires no task-specific or user-specific tuning. The absence of tuning to the task allows us to hope it might generalize more than some highly fit model of workload. Finally, rather than documenting a relationship between TARP and difficulty, we close the loop and focus on the effect of our neuro-adaptive training approach on training outcomes. Our developed technology has potentially wide relevance, as it can be applied to virtually any computer-based human learning experience. However, more work is required to characterize the generalizability of the combined behavioral and neural training approach. Different training contexts will likely require adjustments to the parameters of the adaptive rules. Moreover, a CAT applied to other training contexts might benefit from additional or different neural or other physiological measures.

Although behavioral adaptive training has had notable success, including neural signals for adaptation provides additional insight. The benefits of our novel approach are broad within the context of interactive training (e.g., game-based cognitive training) and could allow for improved transfer performance, reduced training times, and reduced training costs.

**Applications**. Neuroadaptive technology can be used in many areas (Fafrowicz et al., 2012), such as decision support, human-robot teams, learning, and memory. We observed performance improvement in a stimulus recognition transfer task, following CAT training in a go/no-go inhibitory control task. Future work should examine how robust this finding is to other training and transfer tasks, and how robust this finding is when compared to other, perhaps more sophisticated, forms of BAT. The general approach of incorporating TARP into adaptive training systems could be applied to existing adaptive training of perceptual or cognitive tasks in which blocks of practice are subject to an adaptive difficulty modification (e.g., tutoring, exercise, simulated training). Virtual reality (VR) based adaptive training could be a particularly fruitful area to explore, because it needs to satisfy similar constraints. Our system uses laboratory-grade EEG, but the same principle could be applied using consumer-grade EEG. The incorporation of EEG into Virtual Reality (VR) headsets opens the door for combined adaptive training to be used in VR. This substantially expands the range of applications for our work. Whether adaptive training takes place with conventional or VR hardware, the addition of neural measures to such systems might result in better learning.

## 5. Conclusion

We have explored a novel approach of designing an adaptive training by incorporating both behavior and a neural measure (Theta/Alpha Ratio Percentage, TARP) as the performance metrics feeding the adaptive logic. We evaluated this newly designed adaptive training using an abstract threat/non-threat training task and a contextualized transfer task. We found that participants trained with the CAT system had higher accuracy in the transfer task compared to those trained with the other two systems. The experimental results showed that CAT improvement was 6% relative to SIFD and 9% relative to the BAT system. Future work can determine whether these results could be replicated in a revised behaviorally adaptive system, or if these advantages are uniquely attributable to the inclusion of the neural measure.

## Data Availability Statement

The datasets presented in this study can be found in online repositories. The names of the repository/repositories and accession number(s) can be found at: https://osf.io/pum6n/.

## Ethics Statement

The studies involving human participants were reviewed and approved by DEVCOM ARL Human Research Protections Program under protocol 17-166. The patients/participants provided their written informed consent to participate in this study.

## Author Contributions

MR, BF, AO, KP, PK, CS, and AP contributed to conception and design of the study. MR and AP implemented the adaptive training. MR and AO collected data. MR, BF, and KP conducted statistical analyses. MR, BF, AO, and KP wrote sections of the manuscript. All authors contributed to manuscript revision, read, and approved the submitted version.

## Funding

This work was supported by DEVCOM ARL's Human Sciences Campaign.

## Conflict of Interest

AO was employed by DCS Corporation. The remaining authors declare that the research was conducted in the absence of any commercial or financial relationships that could be construed as a potential conflict of interest.

## Publisher's Note

All claims expressed in this article are solely those of the authors and do not necessarily represent those of their affiliated organizations, or those of the publisher, the editors and the reviewers. Any product that may be evaluated in this article, or claim that may be made by its manufacturer, is not guaranteed or endorsed by the publisher.
